# Data set on the antibacterial effects of the hydro-alcoholic extract of *Ferula assafoetida* plant on *Listeria monocytogenes*

**DOI:** 10.1016/j.dib.2018.08.057

**Published:** 2018-08-28

**Authors:** Maliheh Akhlaghi, Morteza Abbasi, Yahya Safari, Reza Amiri, Nasrin Yoosefpour

**Affiliations:** a Student of Environmental Health Engineering, Health Faculty, Students Research Committee, Gonabad University of Medical Sciences, Gonabad, Iran; b Behvarz Training Center, Torbat Heydariyeh University of Medical Sciences, Torbat Heydariyeh, Iran; c Research Center for Environmental Determinants of Health, Kermanshah University of Medical Sciences, Kermanshah, Iran; d Students Research Committee, School of Public Health, Kermanshah University of Medical Sciences, Kermanshah, Iran

**Keywords:** Antibacterial, *Ferula assafoetida*, *Listeria monocytogenes*, Hydro-Alcoholic Extract

## Abstract

The aim of the collection of the present dataset is to show the antibacterial effects of the hydro-alcoholic extract of *Ferula assafoetida* plant on *Listeria monocytogenes*. Firstly, the *Ferula assafoetida* herb was collected from investigated hills in Gonabad, Khorasan province, Iran. After botanical and pharmocognosy investigation, the hydro-alcoholic extract of this herb was produced using percolation method. Then, its antimicrobial effects, Minimum Inhibitory Concentration (MIC) and Minimum Bactericidal Concentration (MBC), were investigated using Disc Diffusion Method and Macro Dilution Method vs. Listeria Monocytogenes, serotypes 4a and 4b, respectively. Also, Ampicillin (10 µg/disc) was used as a reference antimicrobial material. The data showed that MIC and MBC of the *Ferula assafoetida* extract on *Listeria monocytogenes* was 7.25 µg/ml and 12.50 µg/ml, respectively. Moreover, the average diameter of the inhibitory zone of extracted hydrolcoholic from Ferula Alliacea plant over Listeria monocytogenes 4a and 4b (30 g/ml) were 16.35 2.5 and 15.87 3.1 mm, respectively, and in order for ampicillin was 24.31 2.45 and 24.31 2.45 mm. The hydro-alcoholic extract produced from *Ferula assafoetida* can be used as the antibacterial for *Listeria Monocytogenes.*

**Specifications Table**

**Table:** 

Subject area	Environmental health science
More specific subject area	Medicine
Type of data	Tables
How data was acquired	The *Ferula assafoetida* herb was collected from the investigated hills in Gonabad, Iran. The hydro-alcoholic extract of this herb was produced using percolation method. It׳s antimicrobial effects, Minimum Inhibitory Concentration (MIC) and Minimum Bactericidal Concentration (MBC) were measured using Disc Diffusion Method and Macro Dilution Method versus Listeria monocytogenes, serotypes 4a and 4b, respectively
Data format	Raw, analyzed
Experimental factors	In each test series, a disk containing 15 ml of DMSO (Dimthylsulfoxide) and methanol as negative control and a disk containing μg/disc 10 Ampicillin as standard antibiotic were used.
Experimental features	All sampling and antimicrobial effects analysis of the collected samples were performed according to the standard method presented in valid references [1], [2], [3], [4], [5].
Data source location	Gonabad, Iran
Data accessibility	Data are included in this article
Related research article	A. Amalraj, S. Gopi, Biological activities and medicinal properties of Asafoetida: A review, J. Tradit. Complement. Med. 7(2017)347-59 [6].

**Value of the data**

• The obtained data showed that the hydro-alcoholic extract *of Ferula assafoetida* can be an appropriate antibacterial of *Listeria Monocytogenes.*
• Due to the health benefits of *Ferula assafoetida* and its high frequency in Iran, the data of this article could be valuable and encourage researchers to do future similar studies.
• The obtained data can be useful for the investigation of new antibacterial agents for other bacteria in various environments.

## 1 Data

Comparison of the non-growth holes’ diameter of *Ferula Alliacea* extracts vs. serotypes 4b and 4b of Listeria monocytogenes were presented in Table 1. In MIC and MBC dilution tests, the hydro alcoholic extract for both serotype 4a and 4b Listeria monocytogenes was obtained at 7.25 and 12.50 μg/ml, respectively (Table 2, Table 3). The minimum inhibitory concentration (MIC) was 7.25 μg/ml, the minimum mortality concentration (MBC) was 12.50 μg/ml and the diameter of the non-growth holes for Listeria monocytogenes was15.87 ± 3.1 4a mm and for bacteria Listeria monocytogenes observed at 15.87 ± 3.1 4b mm. The results showed that there was no significant difference between Ferula Alliacea extract and two serotypes of Listeria monocytogenes (Table 1, Table 2, Table 3).

**Table 1:** Average diameter of the inhibitory zone of hydrolcoholic extracted from *Ferula Alliacea* plant over *Listeria Monocytogenes* (30 μg/ml).

**Agent**	**Average diameter of inhibition zone (mm)**	
	***Listeria Monocytogenes*****4a**	***Listeria Monocytogenes*****4b**
*Ferula Alliacea*	16.35 ± 2.5 *	15.87 ± 3.1*
Ampicillin	24.31 ± 2.45	20.18 ± 1.80
Evidence	–	–

* Compared with Ampicillin (P <0.01)

**Table 2:** Minimum Inhibitory Concentration of hydrolcoholic extracted from *Ferula Alliacea* plant over *Listeria Monocytogenes* Serotype 4a and 4b.

**Repeat**	**μg/ml**									
	**1**	**2**	**3**	**4**	**5**	**7.25**	**15**	**20**	**Extract control**	**Positive control**
One	+	+	+	+	+	−	−	−	−	+
Two	+	+	+	+	+	−	−	−	−	+
Three	+	+	+	+	+	−	−	−	−	+

**MIC:** Minimum Inhibitory Concentration (−)

Microbial growth or turbidity(+)

**Table 3:** Minimum Bactericidal Concentration of hydrolcoholic extracted from *Ferula Alliacea* plant over *Listeria Monocytogenes* Serotype 4a and 4b.

**Repeat**	**μg/ml**						
	**7.25**	**8**	**9**	**11**	**12.5**	**20**	**Negative control**
One	+	+	+	*+	−	−	−
Two	+	+	+	+	−	−	−
Three	+	+	+	+	−	−	−

* *Listeria Monocytogenes* Serotype 4a not growth

Microbial growth or turbidity (+)

Minimum Bactericidal Concentration (−)

## 2 Experimental design, materials and methods

The serotypes 4a and 4b of Listeria monocytogenes were provided by the Razi Institution (Tehran, Karaj). Linear flow ventilation was used in all microbiological tests which required an aseptic environment. The lyophilized bacterial mice were first transmitted in open aseptic condition and incubated in a BHI (Brain Heart Infusion) fluid medium for 35 h at 35 °C. Then, to ensure the obtained purity of the bacteria, the BHI medium was cultured overnight on a differential-selective Oxford Agar culture medium containing a special supplement, and incubated for 35 h at 35 °C for 48 h. After that, a loop from the typical and black colonies of the bacteria was harvested, priority each test was inoculated with liquid BHI (24-h culture medium) for 24 h to evaluate the antimicrobial effects of each 24-h fresh culture. The investigated amount of 24-h fresh medium was transferred to normal sterile saline tubes using sterilized pipettes, and then the microbial suspension turbidity was prepared using standard McFarland, 0.5 standard solution was approximately 108 1.5 CFU/ml set. The BHI liquid medium was used in tube dissipation experiments and the BHI Agar medium was in disk diffusion experiment. All culture media were prepared according to MERK-Germany׳s instructions and sterilized using the autoclave.

### 2.1 Preparation of hydro-alcoholic extract

The plant used was Ferula from the Alliacea family collected in spring (May) from the foothills in Gonabad (Khorasan province, Iran). Botanical tests were performed according to Iranian Pharmacopoeia in Pharmacognosy Department, Mahshad University of Medical Sciences. After collection, the shoot was isolated and fully dried and crushed for extraction. Extraction was performed by percolation method using 80% ethanol solvent. The ratio of used solvent content was determined and used for extracting 10 to 1 plants [4]. After the extraction, the extracts were concentrated by vacuum distillation at a temperature of about 40 °C and continued to reach about 5% of the initial amount of each extract. Concentrated extracts were prepared by solvent DMSO (Dimthylsulfoxide) and methanol with ratios of 60 to 40 dilutions to apply in MIC and disk diffusion tests [5].

### 2.2 Investigation of antimicrobial effects

The minimum inhibitory concentration (MIC) and minimum inhibitory concentration of antimicrobial agent (MBC) were determined using a tube dilution method [6]. To determine MIC, a series of 10 test tubes were used for each extract. 8 tubes to test different dilutions of each extract were used, and a tube as a positive control and a tube as a negative control was used. Each extract was diluted in tube number 1 at a concentration of 20 μg/ml, and tube number 8 at a concentration of 1 μg/ml in a culture medium of BHI of the broth with 1 ml of microbial suspension containing 1.5 × 108 CFU/ml bacterium. A tube containing 9 ml of culture medium plus 1 ml of diluted extract as a positive control and a tube containing 9 ml of culture medium plus 1 ml of bacterial suspension as negative control was prepared. All test tubes were placed at 35 °C for 24 h. After incubation, the tubes were examined due to the cloudy led by inoculated bacterial growth. This method was repeated 3 times for each Listeria Monocytogenesis serotype. Samples were taken from all tubes in which there was no growth of the bacterium. The bacteria were cultured using Pour Plate Method to determine the minimum concentration of the extract. For this purpose, 1 ml of each tube was mixed with 20 ml of BHI agar mixture at a temperature about 48 °C in Petri dishes, and after agar closure and incubation for 24 h, the culture plates were tested for microbial growth. The tube which was containing the lowest concentration of the extract and the corresponding plate shown the lack of bacterial growth was considered as the MBC of that substance.

To perform disc diffusion experiments, at first a 24-h culture of a suspension bacterium containing 108 bacteria per ml was prepared using McFarland׳s standard solution. To prepare the discs, each disk was saturated with 15 μl of the extract prepared at 30 mg/ml concentrations in each plant. In each test series, a disk containing 15 ml of DMSO and methanol as negative control and a disk containing μg/disc 10 Ampicillin as standard of antibiotic were applied. In these analyses, BHI agar was used as Layer Base and BHI Agar medium plus 1 ml of microbial suspension as a layer containing Layer Base. And then the microbial layer poured into the lower layer and close it, the discs were placed at an appropriate distance from each other. The culture media were stored in a 35 °C oven for 24 h. Then, the diameters of the non-growth holes were measured and the mean was reported [7], [8], [9], [10]. Antimicrobial effects tests were carried out in Microbial Control Laboratory in Pharmacy School, Mashhad University of Medical Sciences, and each test was repeated at least three times. The difference between two bacterial serotypes and control samples were investigated by *t*-test.
